# Feasibility of deep learning-accelerated ultrafast T1-weighted VIBE Dixon imaging of the pelvis for screening of metastases in prostate MRI

**DOI:** 10.1186/s41747-026-00758-3

**Published:** 2026-06-25

**Authors:** Andrea Nedelcu, Maximilian Frederic Russe, Caroline Wilpert, Benedict Oerther, Dominik Marcel Nickel, Ralph Strecker, Fabian Bamberg, Jakob Weiß, Hannes Engel

**Affiliations:** 1 Department of Diagnostic and Interventional Radiology, University Medical Center Freiburg, University of Freiburg, Freiburg im Breisgau, Germany; 2 MR Application Predevelopment, Siemens Healthineers AG, Forchheim, Germany; 3 EMEA Scientific Partnerships, Siemens Healthineers AG, Forchheim, Germany

**Keywords:** Artificial intelligence, Deep learning, Magnetic resonance imaging, Neoplasm metastasis, Prostatic neoplasms

## Abstract

**Objective:**

This study aims to assess the image quality and perceived diagnostic confidence of research deep learning (DL)-accelerated T1-weighted “volumetric interpolated breath‑hold examination” (VIBE) Dixon sequences compared to conventional T1-weighted VIBE Dixon sequences for the screening of pelvic metastases in prostate magnetic resonance imaging (MRI).

**Materials and methods:**

Consecutive patients receiving prostate MRI between February and April 2024 were prospectively included. In addition to the conventional T1-weighted VIBE Dixon sequences (T1STD), two research DL-accelerated sequences (T1DL, T1DL FAST) with different acceleration were acquired. Three blinded radiologists assessed image quality and perceived diagnostic confidence on a Likert scale (from 1 = non-diagnostic to 5 = excellent). Signal homogeneity was measured for quantitative assessment.

**Results:**

Fifty-four patients were included, aged 68 ± 6.6 years (mean ± standard deviation). The DL-accelerated sequences had markedly shorter acquisition time (15 s and 45 s *versus* 143 s). Overall image quality was comparable or superior, with especially improved sharpness in contrast-enhanced imaging (median [interquartile range]: T1STD = 4 [3‒4] *versus* T1DL = 5 [5‒5] *versus* T1DL FAST = 4 [4‒5]; *p* < 0.001). Although a slight increase in artifacts and signal inhomogeneity was noted, the diagnostic confidence was equally excellent with a median score of 5 [5‒5] and with *p* values ranging from 0.276 to < 0.001.

**Conclusion:**

DL-accelerated T1-weighted VIBE Dixon imaging in prostate MRI is feasible and demonstrates good image quality and equally high perceived diagnostic confidence for pelvic metastases with a tremendously shorter acquisition time.

**Relevance statement:**

The availability of prostate MRI is significantly influenced by acquisition time. To meet the increasing demand for prostate MRI, DL-accelerated sequences provide faster and more efficient image reconstruction.

**Key Points:**

- DL-accelerated T1-weighted VIBE Dixon sequences in prostate MRI address the growing demand for rapid, high-quality imaging in prostate cancer assessment.
- DL-acceleration reduces sequence acquisition time from 143 s to 15 s without loss of image quality and perceived diagnostic confidence.
- DL-accelerated T1-weighted VIBE Dixon sequences enable more efficient, time-saving prostate MRI to increase availability and patient throughput.

**Graphical Abstract:**

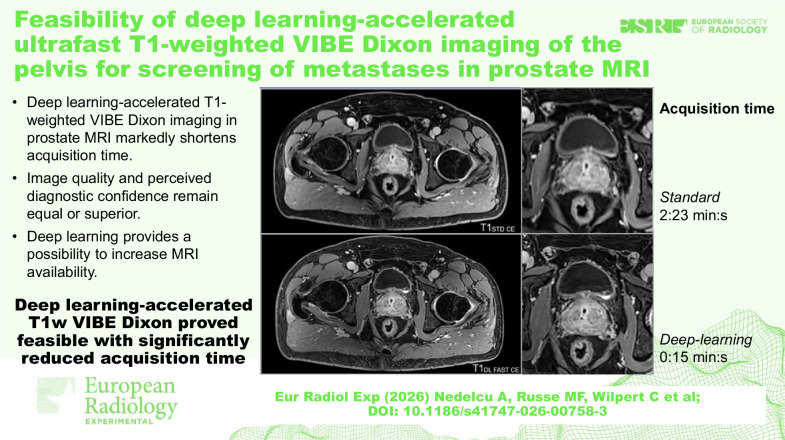

## Background

Magnetic resonance imaging (MRI) is an important tool for detection, risk stratification, active surveillance, and local staging of prostate cancer [1]. In men, prostate cancer is the world’s second most common cancer and a leading cause of cancer-associated mortality, with approximately 1.5 million new cases and 397,000 deaths in 2022 [2]. Early detection and accurate staging are crucial for patient outcomes. Demand for prostate MRI is increasing, with numbers rising over recent years [3]. As MRI availability is greatly affected by image acquisition time, the development of faster and more efficient imaging techniques is necessary to meet the growing demand.

According to the current Prostate Imaging Reporting and Data system (PI-RADS) 2.1 guideline, T1-weighted imaging should be obtained for all prostate MRI examinations [4]. It can be useful for the detection of intraprostatic hemorrhage and screening for pelvic metastases, the latter especially with contrast agent [5]. This may enable early detection of metastases, showing especially high specificity for pelvic lymphatic metastases [6].

Using the Dixon technique for the T1-weighted sequence is particularly advantageous in the characterization of bone lesions, as it provides superior fat suppression and enhanced contrast resolution [7]. By clearly delineating fat and water content within lesions, the Dixon technique improves the differentiation between benign and malignant bone pathologies. The verification of intraprostatic hemorrhage on non-contrast T1-weighted imaging is of great importance, as the resulting restriction in the diffusion-weighted imaging (DWI) sequence may otherwise lead to false positive findings and possibly even unnecessary biopsies [8].

Acquisition of conventional multiparametric MRI sequences, such as T1-weighted imaging, can be time-consuming, limiting the efficiency of the examination and potentially reducing patient comfort and compliance. Over time, several acceleration techniques have been developed that significantly reduce MRI acquisition times, such as parallel imaging, which has been in use for approximately two decades now [9]. Recently, deep learning (DL) algorithms have shown great potential in further accelerating MRI examinations, while maintaining or even improving image quality [10]. DL-based reconstruction algorithms can learn the complex nonlinear mapping between undersampled and fully sampled MRI data, enabling the reconstruction of high-quality images from heavily undersampled k-space data [11]. This approach has been successfully applied to various MRI sequences and anatomical regions, including the brain [12, 13], the breast [14], the knee [15], and the liver [16].

The aim of this study is to compare novel, prospectively accelerated, research DL-reconstructed T1-weighted “volumetric interpolated breath‑hold examination” (VIBE) Dixon sequences with conventional and accelerated sequences with respect to image quality and perceived diagnostic confidence. The study is designed as a feasibility evaluation focusing on image quality and reader confidence, rather than a formal assessment of sensitivity or specificity for the detection of lymph node or bone metastases. We hypothesized that DL-accelerated T1-weighted VIBE Dixon imaging is feasible in clinical routine with image quality and perceived diagnostic confidence comparable or even superior to the standard sequence, while significantly reducing acquisition time.

## Methods

### Study population

The study was approved by the local institutional review board (ethics committee vote number 22-1185), and written informed consent was obtained from all participants. This prospective study included consecutive patients, who were referred to our institution for multiparametric MRI from February to April 2024, for clinical suspicion of prostate cancer or assessment of known prostate cancer without a history of surgical treatment. Indications for MRI included suspicious digital rectal examination, prostate-specific antigen (PSA) elevation > 4 ng/mL or PSA increase of 0.3‒0.7 ng/mL per year. Exclusion criteria were severe motion artifacts in the majority of sequences or incomplete MRI examination. Clinical data (PSA, prostate volume, and body weight) were obtained from radiology reports or hospital information system documents. The PSA value was missing for ten patients.

### MRI protocol

All patients received a multiparametric MRI in accordance with PI-RADS version 2.1 at 3 T (Magnetom Vida, Siemens Healthcare) using an 18-channel body coil. Intravenous butylscopolamine was administered if no contraindications were present. The imaging protocol included T2-weighted imaging, DWI, and dynamic contrast-enhanced (CE) imaging, as recommended by the PI-RADS v2.1 guidelines [4]. In addition to the conventional T1-weighted VIBE Dixon sequence (T1_STD_), two protocol versions of a research DL-accelerated T1-weighted VIBE Dixon sequence (T1_DL_ and T1_DL FAST_) with different acceleration were acquired. T1STD was acquired with a total acceleration factor of 3 and 3 averages, resulting in an acquisition time of 143 s. T1_DL_ and T1_DL FAST_ were acquired with a total acceleration factor of 4 and 6, each with one average, resulting in an acquisition time of 45 s (T1_DL_) and 15 s (T1_DL FAST_). T1_STD_, T1_DL_, and T1_DL FAST_ were obtained before and after intravenous administration of a gadolinium-based contrast agent (0.1 mmol/kg body weight; Gadovist, Bayer Healthcare), resulting in six different sequences investigated in this study.

All T1-weighted VIBE Dixon sequences were acquired with a slice thickness of 2 mm, a field of view of 400 × 300 mm^2^ covering the entire pelvis, using Controlled Aliasing in Parallel Imaging Results in Higher Acceleration (CAIPIRINHA) [17], a flip angle of 9°, an anterior-posterior phase encoding direction, a distance factor of 20%, and a slice oversampling of 34.5%. The sequence parameters are listed in Table 1.

**Table 1:** MRI parameters

Parameters	Standard	DL	DL FAST
Acquisition time [s]	143	45	15
Number of excitations	3	1	1
Acceleration factor	3	4	6
Repetition time [ms]	5.4	4.5	4.2
Echo time [ms]	2.46/3.69	1.47/2.48	1.41/2.81
Bandwidth [Hz/Pixel]	800	920	930
Voxel size [mm^3^]	0.9 × 0.9 × 2.0	0.5 × 0.5 × 2.0	0.5 × 0.5 × 2.0
Acquisition matrix	448	416	384
Phase resolution [%]	90	90	70
Slice resolution [%]	80	80	72
Phase oversampling [%]	0	25	25
Slice oversampling [%]	35.4		
Fat-water contrast	Dixon		
Reconstruction	CAIPIRINHA	DL-enhanced CAIPIRINHA	DL-enhanced CAIPIRINHA
Interpolation mode	‒	DL super-resolution	DL super-resolution

Acquisition parameters of the T1-weighted VIBE Dixon sequences: standard sequence (Standard), deep learning-accelerated to 45 s (DL), and deep learning-accelerated to 15 s (DL FAST)

*CAIPIRINHA* controlled aliasing in parallel imaging results in higher acceleration

### DL-enhanced image reconstruction

The water-fat separated images were obtained in three sequential steps. First, images of the individual contrasts were generated from k-space data on the acquired resolution using an unrolled network architecture inspired by variational networks [18]. The algorithm receives undersampled k-space data, as well as precalculated coil sensitivity maps as input, and then performs six iterations that alternate between parallel imaging-based data consistency and neural network-based image enhancement. The data consistency step and the parameters of the neural networks were considered trainable and determined through supervised training using pairs derived from about 500 fully sampled 3D datasets acquired from healthy volunteers on 1.5-T and 3-T scanners (MAGNETOM scanners, Siemens Healthineers) in the head, abdomen, and pelvis region. The obtained parameters were then exported for prospective use in the reconstruction pipeline of the scanner as previously employed and detailed for liver imaging [16, 19]. In the second step, the combined, complex-valued images were interpolated by a factor of 2 in all spatial dimensions using a DL-based super-resolution algorithm that was trained in a supervised manner for the selected partial Fourier sampling as outlined in prior studies [16, 19, 20]. Similar to the reconstruction from k-space, determined model parameters were exported for prospective use on the scanner. In the final, third step, the water and fat images were then derived from the calculated CE images using the conventional, scanner-integrated Dixon algorithm.

### Image analysis

Qualitative analysis: Three readers (radiologists with 3, 5, and 6 years of experience in prostate MRI) independently assessed image quality and perceived diagnostic confidence across all examined T1-weighted sequences (T1STD, T1DL, and T1DL FAST)—both before and after contrast administration.

Median values and interquartile ranges [IQR, 25th–5th percentiles] for each parameter were assessed. The readers were blinded to the type of sequence and the patient’s clinical data. To minimize potential recognition bias related to the appearance of the standard and DL sequences, all sequences and anonymized patient images were presented in random order to the readers. No prior training or familiarization session with DL images was conducted beforehand. Images were evaluated in random order using a 5-point Likert scale for the following criteria: overall image quality [1: nondiagnostic, 2: poor but still interpretable, 3: fair, 4: good, and 5: excellent], sharpness [1: very severe blurring (nondiagnostic), 2: severe blurring (affecting diagnosis), 3: moderate blurring, 4: minimal blurring, and 5: no blurring], as well as subjective noise and artifacts [1: severe, 2: moderate, 3: mild; 4: minimal; and 5: none]. Artifacts were evaluated based on an overall artifact level, encompassing motion artifacts, aliasing artifacts, ghost artifacts, banding artifacts, and truncation artifacts. Additionally, the readers assessed their perceived diagnostic confidence regarding the detection of suspicious lymph nodes and bone lesions, as well as intraprostatic bleeding in the non-contrast sequences, using also a 5-point Likert scale [1: nondiagnostic, 2: poor; 3: fair, 4: good; and 5: excellent].

Quantitative analysis: For quantitative assessment, signal homogeneity was measured by placing a region of interest of 1 cm radius in size in the piriformis muscle and calculating the coefficient of variation (CV), defined as the ratio of the standard deviation to the mean. The piriformis muscle is suitable for signal homogeneity measurement, since it is less often affected by fatty degeneration and therefore usually provides a fairly homogeneous tissue signal. Additionally, it is also located inside the pelvis, distant from the margins of the image, which are more likely to harbor artifacts, and it is usually distant from susceptibility artifacts.

In addition to the CV, further texture parameters were extracted from the region of interest, including mean signal intensity ± standard deviation, entropy, skewness, and kurtosis. Entropy quantifies the complexity or randomness of the signal distribution, with higher values indicating greater texture heterogeneity. Skewness depicts the asymmetry of the signal intensity distribution, where values near zero indicate a symmetric distribution, positive values indicate a right-skewed distribution, and negative values indicate a left-skewed distribution. Kurtosis measures the “tailedness” of the distribution, with higher values indicating more extreme outliers.

### Statistical analysis

Normality of continuous variables was assessed using the Shapiro–Wilk test. Non-parametric variables and non-normally distributed variables were reported as median and IQR values, whereas variables with an approximately normal distribution were reported as mean ± standard deviation. Image quality scores and perceived diagnostic confidence levels of the three MRI sequences obtained by the three readers were compared using the Friedman test as a suitable non-parametric repeated-measures test to assess overall differences between related samples. When multiple comparison analyses showed significant differences between the three different sequences, a Wilcoxon signed-rank test post hoc paired comparison was performed with Bonferroni correction. For CV analysis, ANOVA with post- hoc pairwise comparisons using Tukey’s Honestly Significant Difference was used for normally distributed data (non-contrast sequences), and the Friedman test for overall comparison of the three sequences, followed by pairwise Wilcoxon signed-rank tests with Bonferroni correction for multiple comparisons was used for non-normally distributed data (CE sequences). Values of *p* < 0.05 were considered statistically significant.

Interrater reliability was assessed using the intraclass correlation coefficient (ICC). A two-way mixed-effects model assessing consistency for single measures (ICC(3,1)) was used, as described by Shrout and Fleiss [21]. ICC values were interpreted as poor (< 0.40), fair (0.40–0.59), good (0.60–0.74), and excellent (≥ 0.75), according to Cicchetti [22].

Non-inferiority of the DL-accelerated sequences (T1_DL_ and T1_DL FAST_) *versus* the standard sequence (T1_STD_) was assessed for diagnostic confidence endpoints using a pre-specified non-inferiority margin of Δ = 0.5 Likert units, representing 10% of the 5-point Likert scale range. For each comparison, the paired Hodges-Lehmann estimator was calculated with bootstrap 95% confidence intervals (10,000 resamples). Non-inferiority was established if the lower bound of the 95% CI exceeded −Δ and the one-sided Wilcoxon. Values of *p* < 0.05 were considered statistically significant.

Due to the focus on feasibility and the limited number of participants with metastases, which is not eligible for statistical significance testing, the study does not aim to assess sensitivity and specificity for lymph node or bone metastases. Statistical analyses were performed using Python using the SciPy package [23]. Python code was partially developed with the assistance of a large language model (Claude Opus 4.5, Anthropic). All code and results were verified by the authors. Graphs were created using the packages matplotlib [24] and seaborn [25].

## Results

A total of 57 men were included in the study. Three patients were excluded due to incomplete acquisition of the research sequences, resulting in 54 patients in the final analysis. The age was 68 ± 6.6 years (mean ± standard deviation), PSA 8.0 ± 4.3 ng/mL, PSA density 0.15 ± 0.12 ng/mL², and body mass index 25.6 ± 3.1 kg/m². One patient had a unilateral hip prosthesis.

Indications for multiparametric prostate MRI included in the study were cancer detection (*n* = 40), primary staging (*n* = 8), and active surveillance (*n* = 6). No patients with biochemical recurrence or prior pelvic radiotherapy were included.

**Fig. 1:**
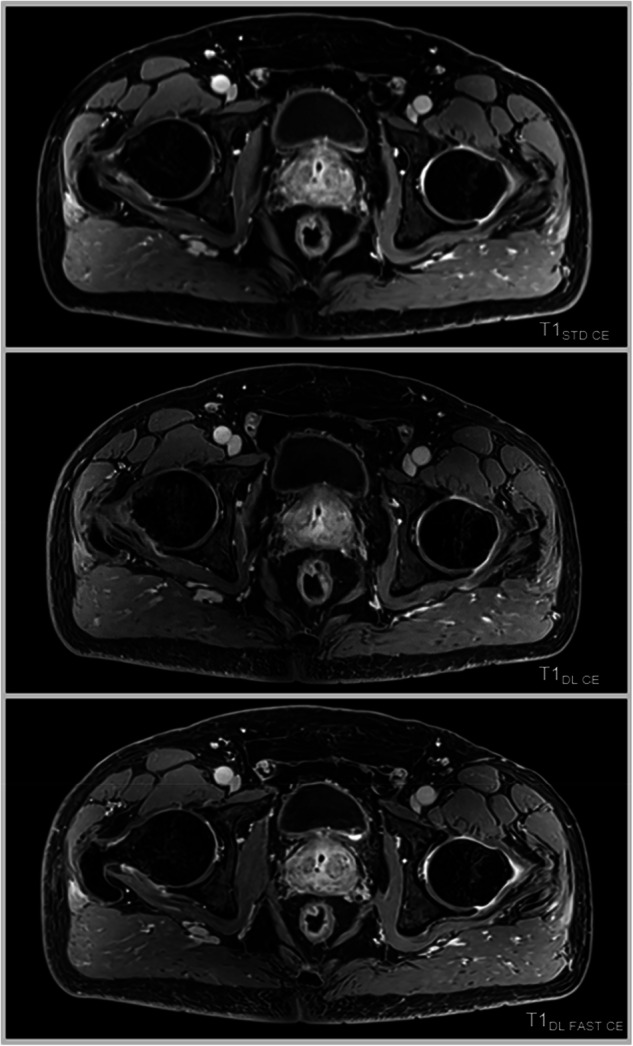
A 70-year-old patient with a PSA of 11.3 ng/mL and suspicion of prostate cancer. CE T1-weighted sequences with water-only images are shown. While the standard sequence (T1_STD CE_) shows good image quality, both research deep learning-accelerated sequences (T1_DL CE_ and T1_DL FAST CE_ with 45-s and 15-s acquisition time, respectively) exhibit improved edge sharpness and overall image quality

Of 21 patients with PI-RADS ≤ 2 index lesion, 6 (29%) received subsequent prostate biopsy (International Society of Urological Pathology [ISUP] grade 0: *n* = 3, ISUP grade 1: *n* = 2, ISUP grade 2: *n* = 1), and 18/19 (95%) patients with PI-RADS ≥ 3 index lesion underwent subsequent prostate biopsy or prostatectomy (ISUP grade 0: *n* = 5, ISUP grade 1: *n* = 2, ISUP grade 2: *n* = 4, ISUP grade 3: *n* = 7). In follow-up 6 months after prostate MRI for metastasis evaluation, 7 patients had received radical prostatectomy with lymphadenectomy and positron emission tomography (PET)/CT, 4 patients had only prostatectomy with lymphadenectomy documented in our hospital records, 8 patients had received prostate biopsy and PET/CT, 14 patients had prostate biopsy only, and 21 patients had no further procedure documented, of which 7 were missing. Three patients had lymph node metastasis confirmed by prostatectomy with lymphadenectomy, and one patient had bone metastasis confirmed by PET/CT. An image example of a patient with suspicion of prostate cancer ist shown below in Fig. 1.

### Qualitative assessment of image quality

**Fig. 2:**
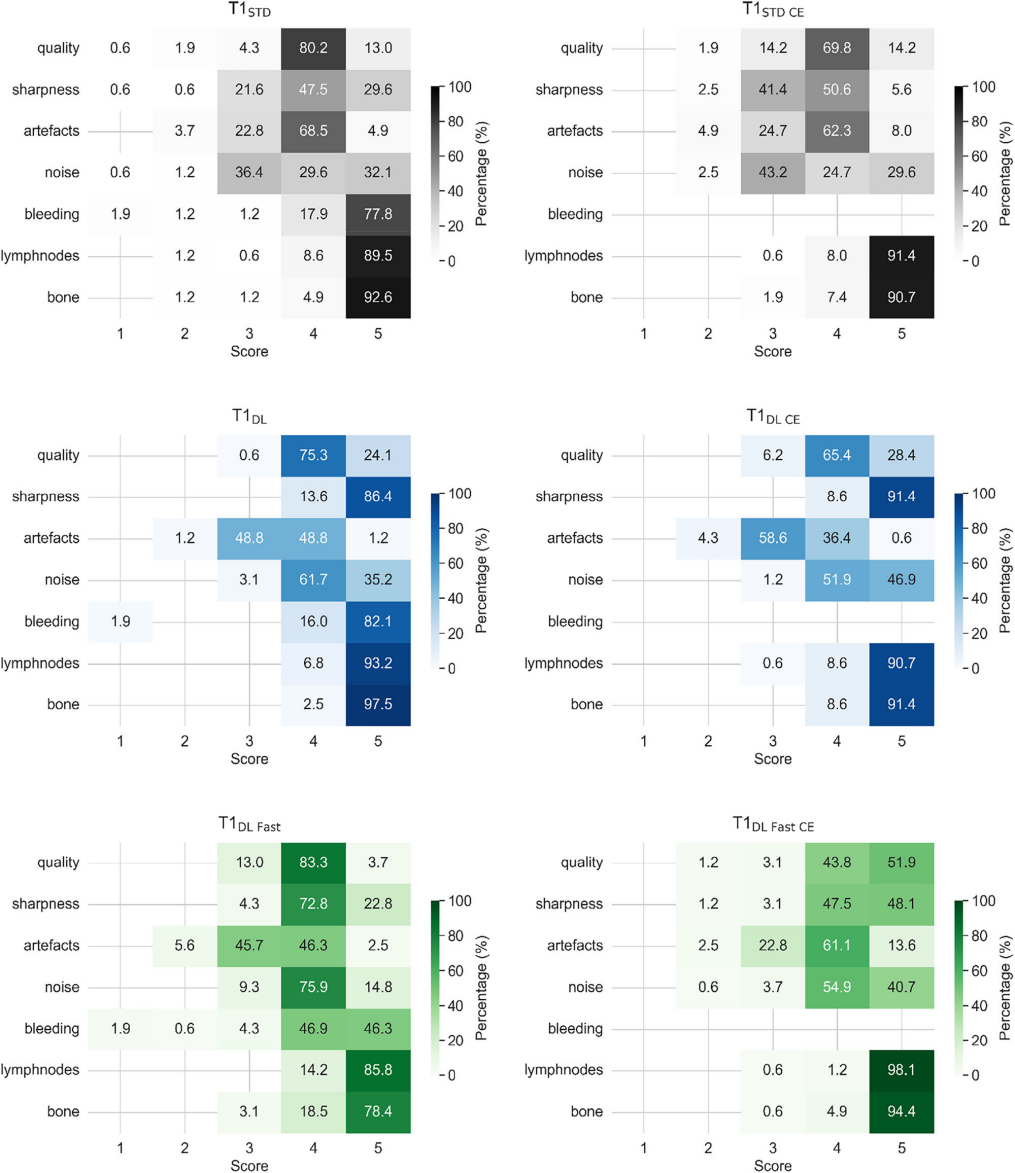
Heatmap showing the percent distribution for each individual item and Likert scale category [1: nondiagnostic; 2: poor but still interpretable; 3: fair; 4: good; 5: excellent] in all examined sequences. Data is listed separately for the standard T1-weighted sequence (T1_STD_, gray) and the research deep learning-accelerated T1-weighted sequences (T1_DL_, blue; T1_DL FAST_, green) with non-contrast sequences on the left and CE sequences on the right

Median values and IQR [25th–75th] for each parameter are summarized in Table 2 and Fig. 2. Table 2 contains *p*-values for statistical significance of pairwise sequence comparison, whereby significance reflects distributional shifts rather than changes in median alone. Overall image quality (on a 5-point Likert scale) was good for all three sequences, with a median of 4 [4‒4] each. Still, slight but statistically significant differences were observed in the distribution of overall image quality: the T1_DL_ scored best, followed by the T1_STD_, and the T1_DL FAST_ sequence scored slightly lower (*p* < 0.001). The T1_DL_ and T1_DL FAST_ demonstrated improved sharpness and reduced noise compared to T1_STD_, though the results were only statistically significant for the T1_DL_ (*p* < 0.001). Especially, sharpness was markedly higher in the T1_DL_, achieving a median score of 5 [5‒5] compared to only 4 [4‒5] for T1_STD_ and 4 [4‒4] for T1_DL FAST_ (*p* < 0.001).

**Table 2:** Comparison of non-contrast sequences

	T1_STD_	T1_DL_	T1_DL FAST_	*p*-value	Significant pairs
Quality	4 [4‒4]	4 [4‒4]	4 [4‒5]	< 0.001	STD‒**DL,** **STD**‒DL FAST, **DL**‒DL FAST
Sharpness	4 [4‒5]	5 [5‒5]	4 [4‒4]	< 0.001	STD‒**DL,** **DL**‒DL FAST
Artifacts	4 [3‒4]	4 [3‒4]	3 [3‒4]	< 0.001	**STD**‒DL, **STD**‒DL FAST
Noise	4 [3‒5]	4 [4‒5]	4 [4‒4]	< 0.001	STD‒**DL,** **DL**‒DL FAST
Bleeding	5 [5‒5]	5 [5‒5]	4 [4‒5]	< 0.001	**STD**‒DL FAST, **DL**‒DL FAST
Lymph nodes	5 [5‒5]	5 [5‒5]	5 [5‒5]	0.091	None
Bone	5 [5‒5]	5 [5‒5]	5 [5‒5]	< 0.001	STD‒**DL,** **STD**‒DL FAST, **DL**‒DL FAST

Median and IQR [25th‒75th percentiles] of the accumulated ratings for image quality and perceived diagnostic confidence assessed by all three readers for the non-contrast standard (T1_STD_) and research DL-accelerated T1-weighted sequences (T1_DL_, T1_DL FAST_)

The last column shows significant differences in pairwise comparison with the higher-rated sequence marked in bold. Statistical significance reflects distributional shifts, rather than solely changes in the median

More artifacts were reported in both DL-accelerated sequences compared to the T1_STD_. Readers mainly reported minimal artifacts (Likert scale category 4) in the T1_STD_. However, for the T1_DL_ and T1_DL FAST_, ratings were distributed more equally between mild to minimal artifacts (Likert scale category 3–4), as depicted in Fig. 2. Interestingly, this finding is contrary to the other items, which favor the DL-sequences.

Perceived diagnostic confidence was high among all readers. Excellent evaluation for the detection of lymph nodes and bone metastases was reached, with a median of 5 and an IQR of 5‒5 for all sequences (Table 2). Concerning statistical significance, the T1_DL_ again proved superior for the assessment of bone lesions, even though the data distribution differed only minimally. There was no difference in lymph node assessment between the sequences. Thus, adequate diagnostic confidence was provided by all examined protocols. Conversely, evaluation of intraprostatic hemorrhage showed a slightly different pattern. Although the median values across sequences remained high, significant differences emerged (*p* < 0.001) with T1_DL FAST_ scoring a slightly lower median of 4 [IQR 4‒5], meaning good evaluation of intraprostatic hemorrhage. T1_STD_ and T1_DL,_ though both scored a median of 5 [IQR 5‒5], meaning excellent evaluability. Nonetheless, these differences did not reach a level suggesting diagnostic impairment.

Median values and IQR for CE imaging are listed in Table 3, the data distribution in greater detail is visualized in Fig. 2. The *p*-values for statistical significance of sequence comparison in Table 3 reflect distributional shifts rather than changes in median alone. For overall image quality, T1_DL CE_ with median 4 [4‒5] and T1_DL FAST CE_ with median 5 [4‒5] both outperformed T1_STD CE_ (*p* < 0.001) with 4 [4‒4], T1_DL FAST CE_ even reached excellent quality. It is apparent that the DL-accelerated sequences especially enhance image sharpness, since this aspect improves the most, compared to T1_STD CE_. Only around 5% of images with T1_STD CE_ are given an excellent score (Likert scale category 5) for sharpness, whereas this is the case for almost 50% in the T1_DL FAST CE_ and over 90% in the T1_DL CE_. Similarly, significantly less noise was observed in the DL- accelerated sequences than in the T1_STD CE_, with no difference between the DL-accelerated sequences.

**Table 3:** Comparison of CE sequences

	T1_STD_ CE	T1_DL_ CE	T1_DL FAST_ CE	*p*-value	Significant pairs
Quality	4 [4‒4]	4 [4‒5]	5 [4‒5]	< 0.001	STD‒**DL**, STD‒**DL FAST**, DL‒**DL FAST**
Sharpness	4 [3‒4]	5 [5‒5]	4 [4‒5]	< 0.001	STD‒**DL**, STD‒**DL FAST**, **DL**‒DL FAST
Artifacts	4 [3‒4]	3 [3‒4]	4 [3‒4]	< 0.001	**STD**‒DL, DL‒**DL FAST**
Noise	4 [3‒5]	4 [4‒5]	4 [4‒5]	< 0.001	STD‒**DL**, STD‒**DL FAST**
Bleeding	5 [5‒5]	5 [5‒5]	5 [5‒5]	0.008	STD‒**DL FAST**, DL‒**DL FAST**
Lymph nodes	5 [5‒5]	5 [5‒5]	5 [5‒5]	0.276	None

The last column shows significant differences in pairwise comparison with the higher-rated sequence marked in bold. Statistical significance reflects distributional shifts, rather than solely changes in the median

Median and IQR [25th‒75th percentiles] of the accumulated ratings for image quality and perceived diagnostic confidence assessed by all three readers for the CE standard (T1_STD_) and research DL-accelerated T1-weighted sequences (T1_DL_, T1_DL FAST_)

The T1_DL CE_ exhibits more artifacts, reaching a median score of 3 (mild artifacts), compared to the T1_DL FAST CE_ and T1_STD CE_, which both scored a median of 4 (minimal artifacts).

**Fig. 3:**
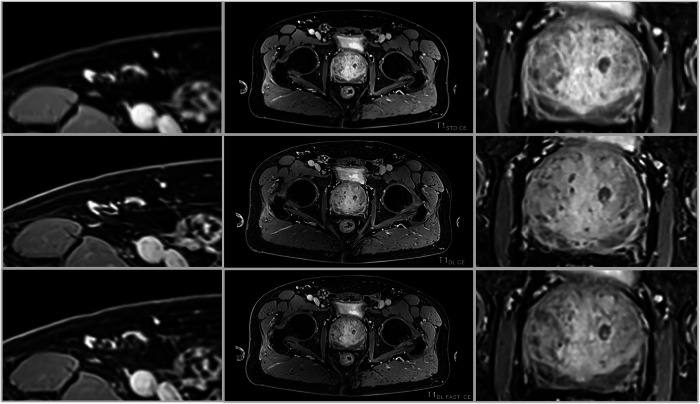
A 63-year-old patient with a PSA of 4.8 ng/mL and suspicion of prostate cancer. Displayed are CE T1-weighted sequences with water-only images and zoomed-in details; on the right, a group of normal right inguinal lymph nodes; on the left, the prostate gland. Standard sequence (T1STD); research DL-accelerated sequences (T1DL with 45-s and T1DL FAST with 15-s acquisition time, respectively)

**Fig. 4:**
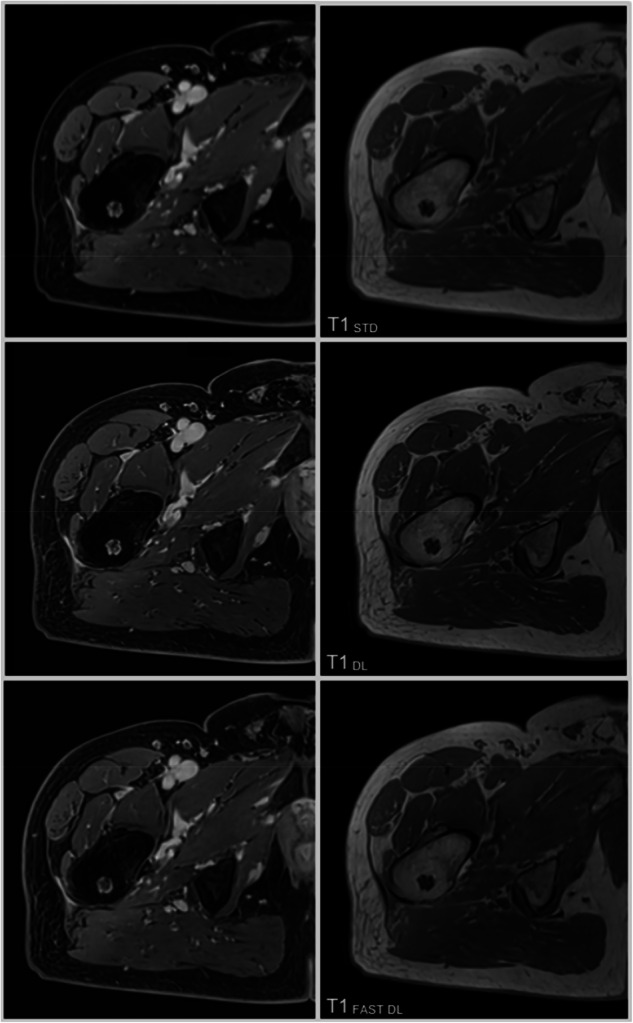
A benign bone lesion, an enchondroma, of the right proximal femur of a 62-year-old patient with a PSA of 6.8 ng/mL and suspected prostate cancer. T1-weighted sequences with contrast- enhanced (CE) water-only images on the right and non-contrast in-phase images on the left are shown. Standard sequence (T1_STD_); research DL-accelerated T1-weighted sequences (T1_DL_ with 45-s and T1_DL FAST_ with 15-s acquisition time, respectively)

Perceived diagnostic confidence in evaluating pelvic lymph nodes and bone lesions remained consistently high across all sequences. While statistical significance was detected in certain comparisons, favoring the T1_DL FAST CE_ for lymph node assessment, all three sequences demonstrated excellent median values of 5 [IQR 5‒5] for lymph nodes and bone lesions (Figs. 3 and 4).

### Quantitative assessment of image quality

**Fig. 5:**
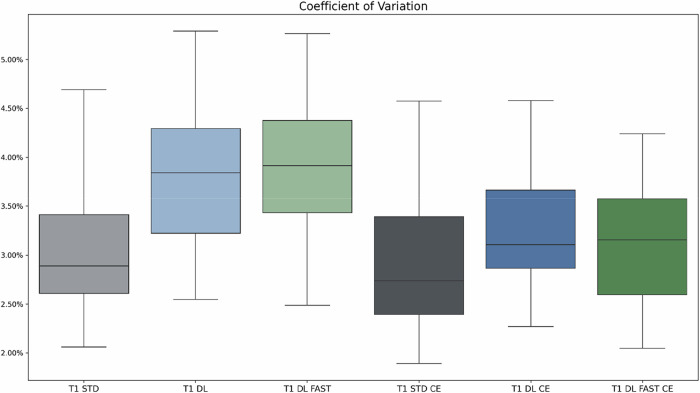
Boxplots of the coefficients of variation for each examined sequence. The line in the middle of the boxes indicates the median, margins of boxes indicate the IQR [25th–75th], and whiskers indicate the total range [0%–100%]. Non-contrast sequences are depicted more transparently on the left, CE sequences on the right. The standard T1-weighted sequences (T1_STD_) show a lower CV (*p* < 0.01), indicating more homogeneous signal, in comparison to the research DL-accelerated T1-weighted sequences = T1DL, T1DL FAST

Analysis of the CV shows relatively low variation of the measured signal intensity values in all sequences, demonstrating an overall homogenous signal in the piriformis muscle. The distribution of the CV values for each sequence is visualized in Table 4 and Fig. 5. For non-contrast T1-weighted imaging, both research DL-accelerated sequences demonstrated a minimally more inhomogeneous signal compared to the T1_STD_, which was statistically significant. No significant difference was found between both DL-accelerated sequences (T1_STD_ *versus* T1_DL_: *p* < 0.001; T1_STD_ *versus* T1_DL FAST_: *p* < 0.001; T1_DL_ *versus* T1_DL FAST_: *p* = 0.809). The same trend was observed for CE T1-weighted imaging. Both DL-accelerated sequences contained a minimally more inhomogeneous signal compared to T1_STD CE_, with no difference between the CE DL-accelerated sequences (T1_STD CE_ *versus* T1_DL CE_: *p* < 0.001; T1_STD CE_ *versus* T1_DL FAST CE_: *p* = 0.045; T1_DL CE_ *versus* T1_DL FAST CE_: *p* = 0.336).

**Table 4:** Quantitative image quality

Non-contrast sequences	Mean	Median	Standard deviation	Minimum	Maximum
T1_STD_	3.06	2.89	0.63	2.06	4.69
T1_DL_	3.80	3.84	0.74	2.55	5.29
T1_DL FAST_	3.89	3.92	0.70	2.49	5.26

CE sequences	Mean	Median	Standard deviation	Minimum	Maximum
T1_STD_	2.89	2.74	0.67	1.89	4.57
T1_DL_	3.25	3.11	0.60	2.27	4.58
T1_DL FAST_	3.12	3.15	0.59	2.04	4.24

Mean, median, standard deviation, minimum, and maximum of values of the CV for the different sequences as percent values

*T1*_DL_ deep learning-accelerated T1-weighted VIBE Dixon sequence, *T1*_DL FAST_ ultrafast deep learning-accelerated T1-weighted VIBE Dixon sequence, *T1*_STD_ standard T1-weighted VIBE Dixon sequence

To further characterize quantitative image properties, additional texture parameters were analyzed. For non-contrast T1-weighted imaging, mean signal intensity differed significantly between all sequences (T1_STD_: 233.5 ± 35.2; T1_DL FAST_: 185.0 ± 29.3; T1_DL_: 194.9 ± 29.8; *p* < 0.001), with DL-accelerated sequences showing systematically lower values. However, the absolute standard deviation within the ROI was comparable across sequences (T1_STD_: 7.15 ± 1.85; T1_DL FAST_: 7.18 ± 1.72; T1_DL_: 7.39 ± 1.80; *p* = 0.486), indicating preserved signal heterogeneity despite the different reconstruction approaches.

Entropy was marginally higher in the DL-accelerated sequences compared to T1_STD_ (3.20 ± 0.23 *versus* 3.29 ± 0.23 and 3.32 ± 0.23 for T1_DL FAST_ and T1_DL_, respectively; *p* = 0.003), suggesting slightly increased texture complexity. The higher-order distribution parameters skewness and kurtosis showed no clinically relevant differences between sequences (skewness: *p* = 0.039; kurtosis: *p* = 0.504).

Similar patterns were observed for CE imaging. Mean signal intensity was significantly lower in DL-accelerated sequences (T1_STD CE_: 275.4 ± 38.7; T1_DL FAST CE_: 238.2 ± 34.0; T1_DL CE_: 239.8 ± 34.3; *p* < 0.001), while standard deviation (*p* = 0.289), skewness (*p* = 0.066), and kurtosis (*p* = 0.929) were comparable across all sequences. Entropy showed a trend toward higher values in DL sequences (*p* = 0.066).

The distribution of texture parameters is summarized in Table 5.

**Table 5:** Additional texture parameters for non-contrast and CE sequences

Non-contrast sequences			
Parameter	T1_STD_	T1_DL_	T1_DL FAST_
Mean signal intensity	233.5 ± 35.2	194.9 ± 29.8	185.0 ± 29.3
Intra-ROI signal standard deviation	7.15 ± 1.85	7.39 ± 1.80	7.18 ± 1.72
Entropy	3.20 ± 0.23	3.32 ± 0.23	3.29 ± 0.23
Skewness	0.04 ± 0.43	0.04 ± 0.34	-0.12 ± 0.32
Kurtosis	0.07 ± 0.83	-0.04 ± 0.61	-0.07 ± 0.54

CE sequences			
Parameter	T1_STD CE_	T1_DL CE_	T1_DL FAST CE_
Mean signal intensity	275.4 ± 38.7	239.8 ± 34.3	238.2 ± 34.0
Intra-ROI signal standard deviation	8.00 ± 2.35	7.79 ± 1.82	7.43 ± 1.76
Entropy	3.29 ± 0.24	3.36 ± 0.22	3.32 ± 0.23
Skewness	-0.05 ± 0.44	0.10 ± 0.52	-0.03 ± 0.42
Kurtosis	0.22 ± 0.95	0.45 ± 1.18	0.27 ± 0.82

Values are presented as cohort-level mean ± standard deviation across cases. The ‘Intra-ROI signal standard deviation’ row refers to the first-order intensity feature describing voxel intensity dispersion within the ROI of each individual case; the ± value therefore reflects between-case variability of this per-case heterogeneity measure. Standard sequence (T1_STD_); research deep learning-accelerated sequences (T1_DL_ with 45-s and T1_DL FAST_ with 15-s acquisition time)

### Interrater reliability analysis

ICC values for the non-contrast sequences demonstrate fair agreement for most items, ranging from 0.41 to 0.51 (overall quality 0.44; sharpness 0.41; bone 0.44, lymph nodes 0.50; artifacts 0.51). Agreement for the evaluation of noise was poor, with an ICC value of 0.20. However, good agreement was reached for assessment of intraprostatic bleeding (ICC value 0.68), which is important, since one of the main purposes of the non-contrast T1-weighted sequence is assessment of intraprostatic hemorrhage.

For the CE sequences, fair agreement was reached for most items (noise 0.40; bone 0.52; overall quality 0.53; artifacts 0.54), good agreement for sharpness with an ICC value of 0.60, and poor agreement for the evaluation of lymph nodes with an ICC value of 0.35.

### Non-inferiority analysis

Non-inferiority analysis confirmed that both DL-accelerated sequences were non-inferior to the standard sequence for diagnostic confidence regarding lymph nodes and bone lesions in both native and CE imaging (all Hodges-Lehmann median differences ≥ -0.167; all lower 95% CI > -0.5; all one-sided *p* < 0.001; Supplementary Table S1). For intraprostatic bleeding assessment in native sequences, T1_DL FAST_ achieved non-inferiority (median difference 0.000, 95% CI [0.000, 0.167]), while T1_DL_ did not meet non-inferiority criteria (median difference -0.333, 95% CI [-0.500, -0.167]), with 8/54 patients (14.8%) showing ≥ 1-point deterioration.

## Discussion

This prospective study underscores the clinical feasibility of DL-accelerated ultrafast T1-weighted VIBE Dixon imaging in patients receiving prostate MRI. The research DL- accelerated sequences, T1_DL_ and T1_DL FAST_, provided equal or enhanced image quality and equally high perceived diagnostic confidence for detecting pelvic lymph nodes and bone metastases. Acquisition time of the DL-accelerated sequences was markedly shorter (T1DL 45 s and T1DL FAST 15 s) compared to the standard T1-weighted VIBE Dixon sequence (T1STD 143 s), representing nearly a 10-fold acceleration without loss of quality. These findings highlight the importance of DL-enhanced imaging techniques and their potential to improve the efficiency of prostate MRI. Shorter acquisition time implies the possibility of increased patient throughput, reduced motion artifacts, and improved patient comfort [26]. Given the growing demand for prostate MRI, accelerated mpMRI acquisition has particular relevance [27].

Formal non-inferiority analysis confirmed that both DL-accelerated sequences were statistically non-inferior to the standard sequence for diagnostic confidence regarding lymph node and bone lesion evaluation using a conservative margin of Δ = 0.5 Likert units. However, T1_DL_ did not meet non-inferiority criteria for intraprostatic bleeding assessment, with 14.8% of patients showing ≥ 1-point deterioration. Interestingly, T1_DL FAST_ achieved non-inferiority for all endpoints despite its shorter acquisition time, possibly due to fewer or different artifacts because the shorter acquisition time of T1_DL FAST_ enables a breath-holding technique. Since bleeding detection primarily serves to prevent false-positive DWI findings and readers typically assess T1-weighted and DWI sequences in conjunction, this limitation may be clinically acceptable; nevertheless, T1_DL FAST_ appears preferable when optimal hemorrhage detection is required.

For almost all aspects of image quality, the three readers scored the DL-accelerated images equal to or significantly better than the standard images. However, the readers reported increased artifacts in both non-contrast DL-accelerated sequences and one of the CE DL-accelerated sequences. Contrary to the T1_DL FAST CE_ was equal to the T1_STD CE_ and did not increase in artifacts. Overall, it seems that the research DL-accelerated sequences produce slightly more artifacts without impairing overall image quality. Additionally, the imaging signal seems slightly more heterogeneous in the DL-accelerated sequences, whereby the IQR considerably overlaps. Despite statistical significance, the absolute numerical difference for the CV was small between standard and DL-accelerated sequences, and these slight changes are unlikely to be perceived by the human eye or yield clinical relevance. Extended texture analysis revealed that while mean signal intensity systematically differed between reconstruction methods, the fundamental signal distribution characteristics were largely preserved. Standard deviation, skewness, and kurtosis showed no significant differences between sequences, indicating that the DL reconstruction maintains the intrinsic texture properties of the imaged tissue. The slightly increased entropy in DL-accelerated sequences may reflect subtle differences in image texture introduced by the DL reconstruction algorithm. However, these differences were small (approximately 0.1 entropy units) and are unlikely to affect diagnostic interpretation. The preservation of signal distribution parameters supports the clinical equivalence of DL-accelerated sequences for quantitative image assessment.

Fair interrater agreement in our study emphasizes that image impression is subjective and may differ to some extent among the readers, regardless of having provided verbal descriptors for each Likert scale category. We did not provide example images for each Likert scale category as orientation for the readers. Providing example images might have improved interrater agreement, but could also interfere with sequence comparison as a confounder. It is noteworthy that one of the readers had less experience than the other two readers (3 years *versus* 5‒6 years), which could partially be responsible for compromising interrater agreement, since the years of experience in reporting prostate MRI are crucial for correct interpretation.

To evaluate the benefit of DL-accelerated T1 imaging in prostate MRI, the effect on the entire examination needs to be taken into account. A standard multiparametric MRI with an acquisition time of 25 min and 45 s at our institution would be reduced by up to 4 min and 16 s (16.6%), when replacing the non-contrast and CE T1_STD_ by the T1_DL FAST_ (2 min and 8 s time reduction per sequence). This results in a total acquisition time of 21 min and 29 s. For the T1_DL_, the total of 25 min and 45 s could be reduced by 3 min 16 s (12.7%) (1 min 38 s per sequence), resulting in 22 min 29 s total acquisition time. With an 8-h scanning day, MRI acquisition time of 25 min and 45 s, and approximately 5 min turnover time between patients, approximately 16 multiparametric MRI examinations can be performed per day. An additional 2 patients could be scanned per day using the T1_DL FAST_, and slightly fewer than 2 patients with T1_DL_.

Raising the acceleration factor and reducing the averages in the DL sequences leads to sparser, undersampled k-space data, a lower signal-to-noise ratio [28, 29], and amplification of aliasing artifacts [30]. Yet, DL reconstruction apparently compensates for this sufficiently to maintain or even improve overall image quality, subjective noise, and particularly sharpness. The increased artifacts of the DL-accelerated images could be due to an increased number of subtle artifacts spread more evenly across the images or the tendency for aliasing to appear toward image edges when using CAIPIRINHA [31]. While DL image reconstruction from undersampled k-space is associated with increased aliasing and banding artifacts, appearing as parallel stripe-like artifacts in regions of decreased signal-to-noise ratio, longer acquisition time in the T1_STD_ is linked to increased motion artifacts. The difference in artifacts and imaging signal homogeneity among the sequences was nonetheless small. The DL-accelerated sequences tend to result in a slightly more artificial or plastic image appearance (Fig. 2), which is also reported by other authors [16, 32]. Wei et al investigated non-contrast and post-contrast DL- enhanced CAIPIRINHA-VIBE sequences for liver imaging with similar acceleration factors as in our study, describing a more synthetic image impression [16]. Although direct comparison is limited due to the different anatomy, they used similar sequence parameters and found enhanced image quality and lesion detection, consistent with our results. Perceived diagnostic confidence for detecting suspicious lymph nodes and bone metastases was excellent for both the standard and the DL-accelerated sequences, supporting their suitability for routine clinical practice. Although studies on DL-accelerated T1-weighted sequences in prostate MRI are lacking, similar results have been reported by Gassenmaier et al for DL-accelerated T2-weighted sequences [33]. They noted increased noise but also superior image quality, sharpness, and diagnostic confidence relative to standard T2-weighted acquisitions. Brendel et al reported comparable findings for DL-accelerated T1-weighted VIBE sequences in the upper abdomen [19]. They achieved improved image quality and no loss of diagnostic confidence at 4-fold and 6- fold acceleration, which aligns with our findings. The diagnostic confidence regarding the evaluation of intraprostatic hemorrhage was slightly limited for the ultrafast DL-accelerated sequence (good *versus* excellent). This might be due to the slightly increased artifacts of the non-contrast ultrafast DL-accelerated sequence (mild *versus* minimal). In general, many studies concerning DL applications in prostate MRI focus on T2-weighted and DWI sequences [33–37]. Research on DL-accelerated T1-weighted sequences in prostate mpMRI is scarce. Hence, comparison with current literature is limited.

Our study has limitations. First, the number of patients (*n* = 54) is modest; thus, the generalizability of the results may be limited. Larger studies are needed to further validate our findings. Second, the study focuses on assessing image quality, rather than diagnostic performance with histopathological correlation or clinical outcomes, and a lesion-based reference standard was not evaluated, so the effect of DL-accelerated T1-weighted VIBE Dixon imaging on subsequent patient management remains unassessed. Although readers were blinded to the type of sequence, they were familiar with the impression of the T1_STD_ from clinical routine, so partial unblinding might have introduced biases. Moreover, the research DL algorithm used in this study is vendor-specific, so the first implementation is expected to be time-consuming, and the findings may not be directly transferable to other manufacturers or platforms. To our knowledge, independent external validation of this algorithm for pelvic imaging has not been performed to date. Finally, this study focused only on T1-weighted VIBE Dixon imaging; the results may not apply equally to other sequences or clinical indications.

Overall, our results indicate that DL-based reconstruction can successfully recover high- quality images from highly undersampled k-space data, substantially reducing acquisition time and therefore enabling ultrafast T1-weighted VIBE Dixon imaging while preserving or even improving image quality and perceived diagnostic confidence.

## Supplementary information

**Additional File 1: Table S1.** Non-inferiority analysis of diagnostic confidence for DL-accelerated T1-weighted VIBE Dixon sequences compared to the standard sequence.

## Data Availability

All data generated and analyzed in this study are available from the corresponding author upon request.

## Abbreviations

CAIPIRINHA: Controlled aliasing in parallel imaging results in higher acceleration
CE: Contrast-enhanced
CV: Coefficient of variation
DL: Deep learning
DWI: Diffusion weighted imaging
ICC: Intraclass correlation coefficient
IQR: Interquartile range
ISUP: International Society of Urological Pathology
MRI: Magnetic resonance imaging
PET: Positron emission tomography
PI-RADS: Prostate imaging reporting and data system
PSA: Prostate-specific antigen
T1_DL_: Deep learning-accelerated T1-weighted VIBE Dixon sequence
T1_DL FAST_: Ultrafast deep learning-accelerated T1-weighted VIBE Dixon sequence
T1_STD_: Standard T1-weighted VIBE Dixon sequence
