# Supplementary material for: Feasibility of deep learning-accelerated ultrafast T1-weighted VIBE Dixon imaging of the pelvis for screening of metastases in prostate MRI
Source: Eur Radiol Exp. 2026 Jun 25;10:98. doi: 10.1186/s41747-026-00758-3 (PMC13304013; doi:10.1186/s41747-026-00758-3)
Supplement: Supplementary file 1 — Additional File 1: Table S1. Non-inferiority analysis of diagnostic confidence for DL-accelerated T1-weighted VIBE Dixon sequences compared to the standard sequence. [file 41747_2026_758_MOESM1_ESM.pdf]

Feasibility of deep learning-accelerated ultrafast T1-weighted VIBE Dixon imaging of the pelvis for screening of metastases in prostate MRI

ELECTRONIC SUPPLEMENTARY MATERIAL

Supplemental Table S1 Non-inferiority analysis of diagnostic confidence for DL-accelerated T1-weighted VIBE Dixon sequences compared to the standard sequence

Non-Inferiority Analysis for Diagnostic Confidence

Comparison	Endpoint	Test median [IQR]	STD median [IQR]	HL difference (95% CI)	*p*-value (non-inf.)	Non-Inf.	n_{det}
$T1_{DL}$ *versus* $T1_{STD}$	Lymph nodes	5.00 [0.25]	5.00 [0.00]	0.000 (0.000, 0.000)	< 0.001	Yes	0/54
$T1_{DL}$ *versus* $T1_{STD}$	Bone	5.00 [0.33]	5.00 [0.00]	-0.167 (-0.167, 0.000)	< 0.001	Yes	3/54
$T1_{DL}$ *versus* $T1_{STD}$	Bleeding	4.50 [0.67]	5.00 [0.33]	-0.333 (-0.500, -0.167)	0.007	No*	8/54
$T1_{DL\ FAST}$ *versus* $T1_{STD}$	Lymph nodes	5.00 [0.00]	5.00 [0.00]	0.000 (0.000, 0.000)	< 0.001	Yes	0/54
$T1_{DL\ FAST}$ *versus* $T1_{STD}$	Bone	5.00 [0.00]	5.00 [0.00]	0.000 (0.000, 0.000)	< 0.001	Yes	0/54
$T1_{DL\ FAST}$ *versus* $T1_{STD}$	Bleeding	5.00 [0.33]	5.00 [0.33]	0.000 (0.000, 0.167)	< 0.001	Yes	0/54
$T1_{DL\ CE}$ *versus* $T1_{STD\ CE}$	Lymph nodes	5.00 [0.00]	5.00 [0.00]	0.000 (0.000, 0.000)	< 0.001	Yes	0/54
$T1_{DL\ CE}$ *versus* $T1_{STD\ CE}$	Bone	5.00 [0.00]	5.00 [0.00]	0.000 (0.000, 0.000)	< 0.001	Yes	1/54
$T1_{DL\ FAST\ CE}$ *versus* $T1_{STD\ CE}$	Lymph nodes	5.00 [0.00]	5.00 [0.00]	0.000 (0.000, 0.000)	< 0.001	Yes	1/54
$T1_{DL\ FAST\ CE}$ *versus* $T1_{STD\ CE}$	Bone	5.00 [0.00]	5.00 [0.00]	0.000 (0.000, 0.000)	< 0.001	Yes	0/54

Non-inferiority was assessed using paired Hodges-Lehmann median differences with bootstrap 95% confidence intervals (10,000 resamples) and one-sided Wilcoxon signed-rank tests. A prespecified non-inferiority margin of $\Delta = 0.5$ Likert units was applied. *CI* Confidence interval, *DL* Deep learning, *HL* Hodges-Lehmann. *Non-Inf.* Non-inferior (lower 95% CI > -0.5 and one-sided $p < 0.05$). n_{det} Patients with ≥ 1-point deterioration, STD Standard (sequence), T1 T1-weighted (sequence). *Lower CI bound equals but does not exceed -Δ (difference).
